# Lessons from the Past: Methodological Issues Arising from Comparison of the Disease Burden of the Influenza A (H1N1) Pandemic 2009–10 and Seasonal Influenza 2010–2019 in the United States

**DOI:** 10.23937/2474-3658/1510218

**Published:** 2021-07-19

**Authors:** James A Koziol, Jan E Schnitzer

**Affiliations:** Proteogenomics Research Institute for Systems Medicine (PRISM), La Jolla, California, USA

## Abstract

**Background:**

Annual influenza outbreaks constitute a major public health concern, both in the United States and worldwide. Comparisons of the health burdens of outbreaks might lead to the identification of specific at-risk populations, for whom public health resources should be marshaled appropriately and equitably.

**Methods:**

We examined the disease burden of the 2009–10 influenza A (H1N1) pandemic relating to illnesses, medical visits, hospitalizations, and mortality, compared to influenza seasons 2010 to 2019, in the United States, as compiled by the Centers for Disease Control.

**Results:**

With regard to seasonal influenza, rates of illnesses and medical visits were highest in infants aged 0–4 years, followed by adults aged 50–64 years. Rates of hospitalizations and deaths evinced a starkly different pattern, both dominated by elderly adults aged 65 and over. Youths aged 0 to 17 years were especially adversely affected by the H1N1 pandemic relative to hospitalizations and mortality compared to seasonal influenza; but curiously the opposite pattern was observed in elderly adults (aged 65 and older).

**Conclusions:**

Determination of a baseline influenza mortality profile in the United States over the 2010–19 decade is not straightforward. The disease burden of the 2009–10 influenza A pandemic among the elderly was strikingly unlike that observed in the subsequent influenza seasons 2010 to 2019: the past did not predict the future.

## Objectives

A reasonable approach to control or mitigate the adverse impact of seasonal influenza and pandemics in the United States would be to focus resources on those subsets of the population most heavily affected by the disease. To that end, we summarize the burden of disease as reflected by illnesses, hospitalizations and mortality associated with the 2009–10 influenza A (H1N1) pandemic, compared to the subsequent influenza seasons 2010 to 2019, as compiled by the Centers for Disease Control. Our goal is to identify specific at-risk populations, for whom public health resources should be marshaled appropriately and equitably.

## Methods

We utilized tables prepared by the Centers for Disease Control (CDC) relating to the estimated influenza disease burden in the United States, from the 2010–11 through the 2019–2020 influenza seasons [1]. Each seasonal table contains estimates of the numbers of symptomatic illnesses, medical visits, hospitalizations, and deaths attributable to influenza, by age groups 0–4 years, 5–17 years, 18–64 years, and 65+ years, with associated estimated ranges [2,3]. In addition, estimated rates of these influenza disease outcomes per 100,000 population in each of these age groups are also provided.

We obtained data relating to the impact of the influenza A (H1N1) pandemic in the United States during the period April 2009 through April 2010 from CDC investigators [4]. These data consist of estimates of illnesses, hospitalizations, and deaths attributable to influenza A, along with ranges, by age groups 0–17 years, 18–64 years, and 65+ years. We converted these counts into rates (per 100000) using 2010 population figures from the US Census Bureau [5]. We remark that the CDC has implemented rigorous protocols [1–4] for data tabulation and presentation, furthering credence in their reports.

To ensure comparability between the influenza A (H1N1) data from 2009–10 and observations in subsequent years, we pooled the age groups 0–4 and 5–17 years in the CDC annual tabulations, and used a random effects beta binomial model [2,6] with linear time trend [7,8] to summarize the weighted average (consensus) disease burden over the period 2010–2020 relating to rates of illnesses, hospitalizations, and deaths for the age groups 0–17 years, 18–64 years, and 65+ years. Calculations were performed in Stata v.14 (StataCorp, College Station, Texas, 2015), as outlined by Guimaraes [9].

## Results

**Figure 1:**
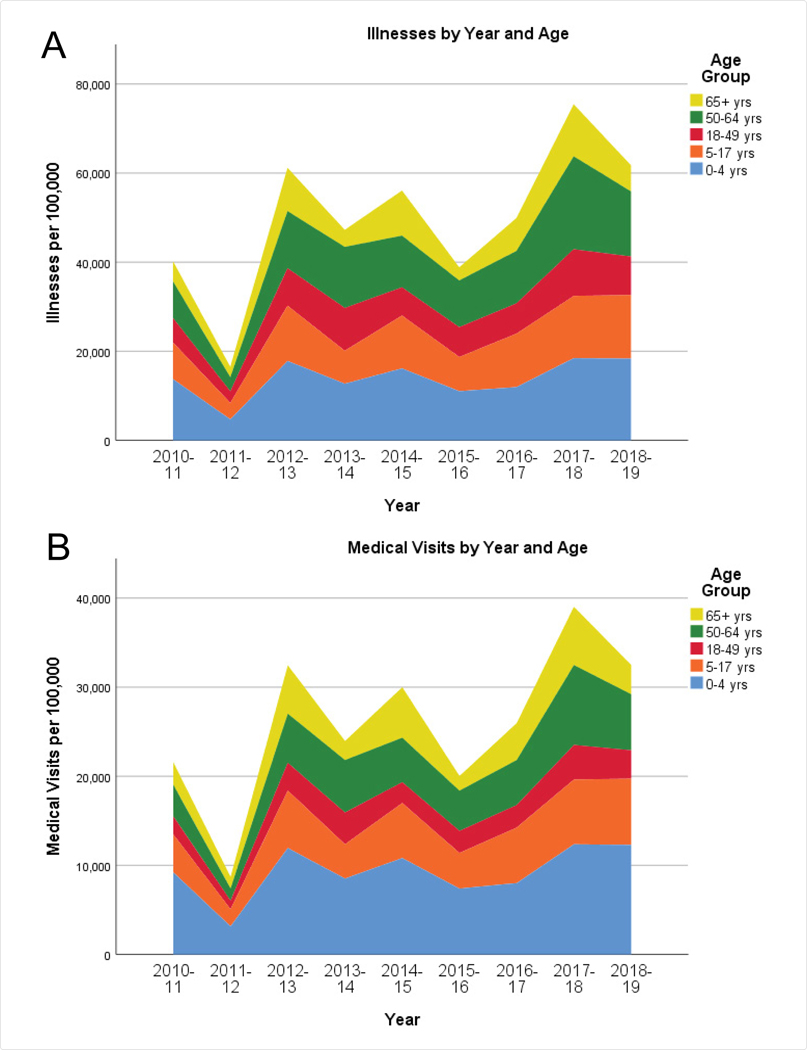

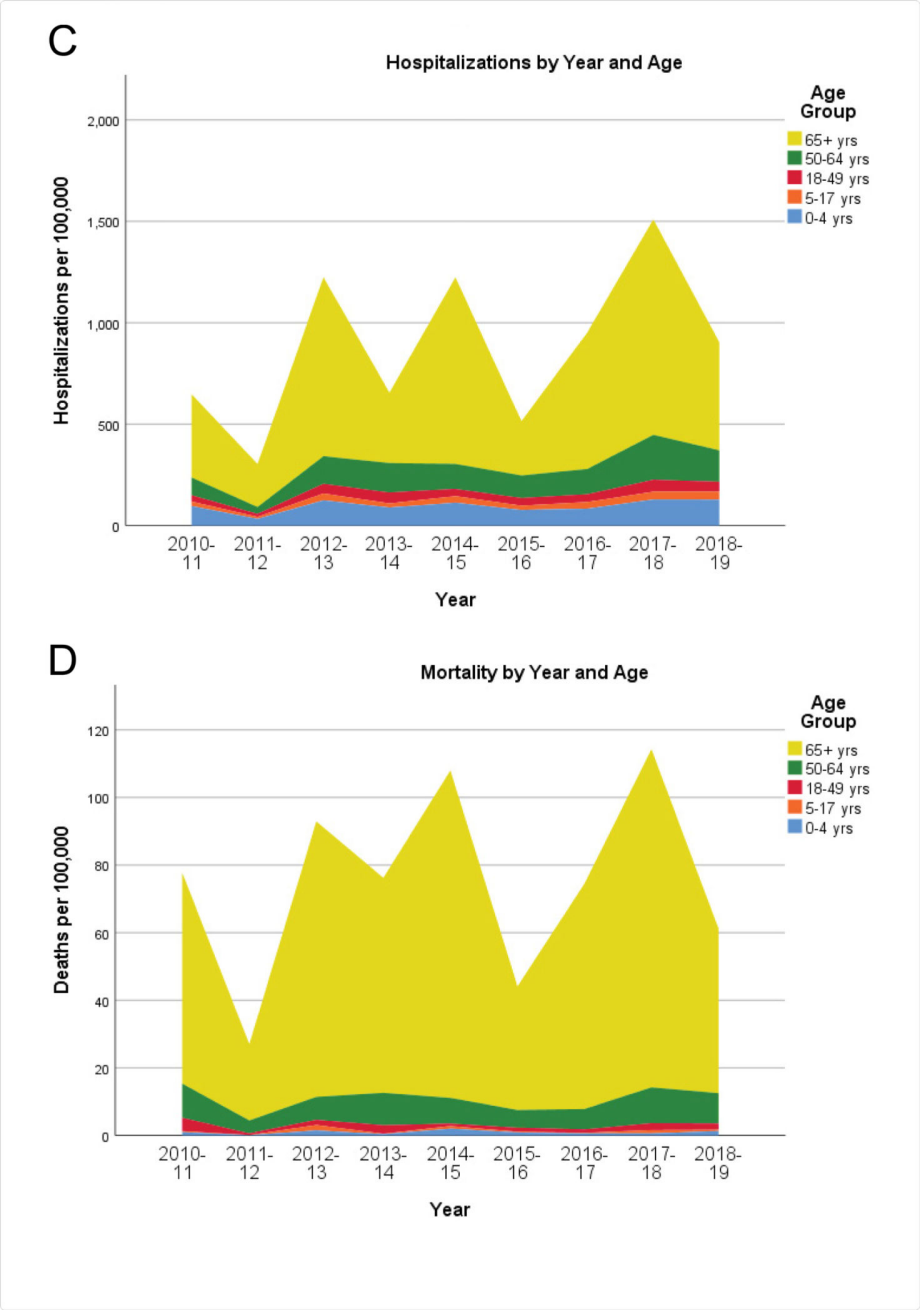
Stacked histograms of illnesses (Figure 1A), medical visits (Figure 1B), hospitalizations (Figure 1C), and deaths (Figure 1D) per 100000 population, by year and age group, 2010–19. Annual observed counts are taken from the Centers for Disease Control website [1], for the entire United States.

In Figure 1, we depict the rates of illnesses, medical visits, hospitalizations, and mortality across the age groups 0–4, 5–17, 18–64, and 65+ attributable to influenza, from the CDC compilations. Rates of illnesses and medical visits are highest in infants age 0–4 years, followed by adults age 50–64 years. Rates of hospitalizations and deaths evince a starkly different pattern, both dominated by elderly adults age 65 and over.

**Figure 2:**
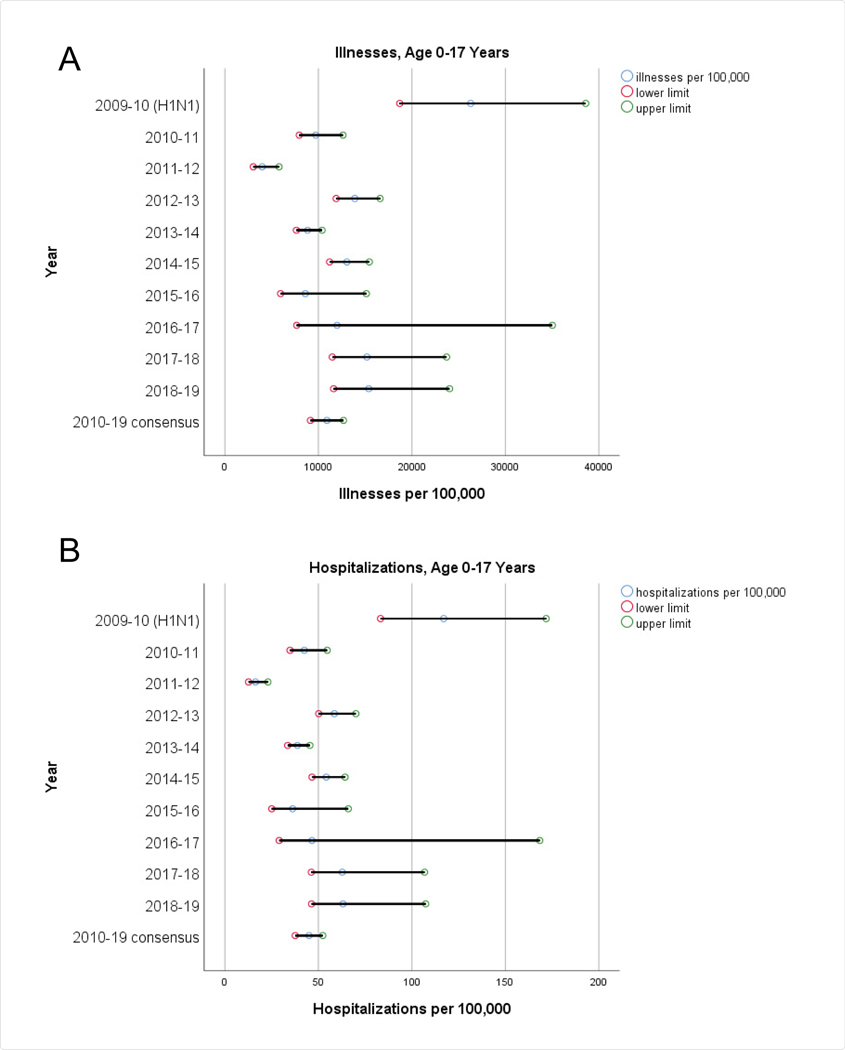

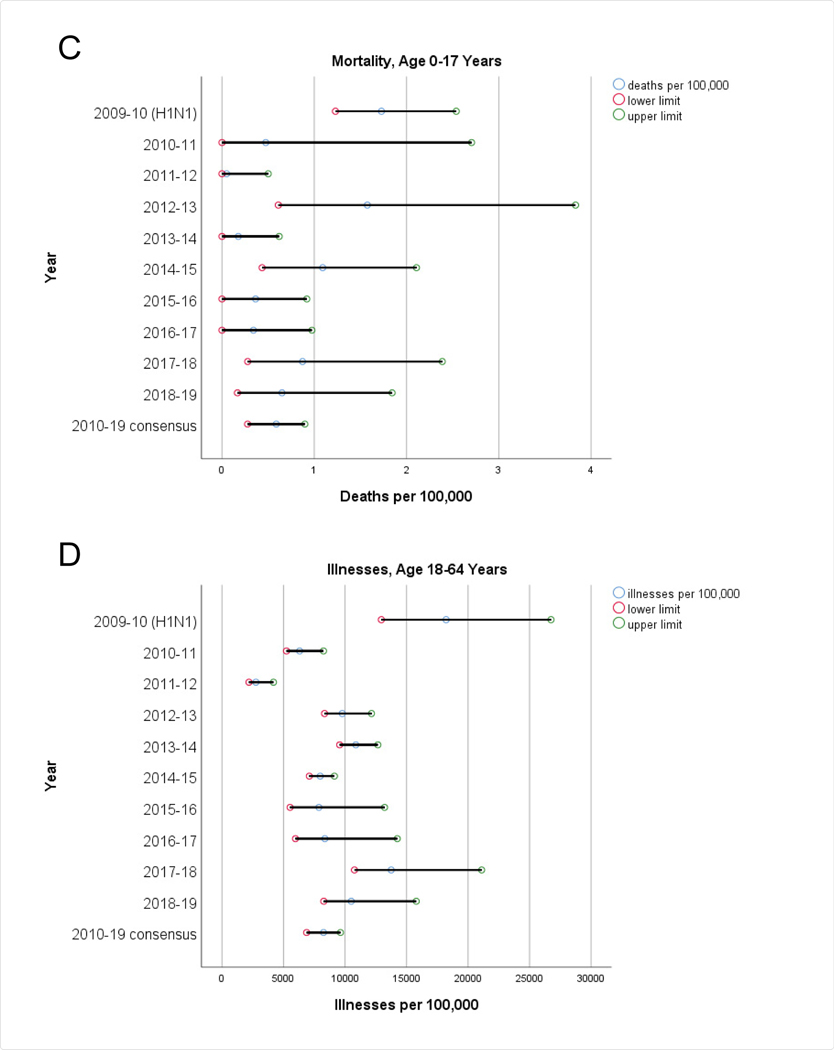

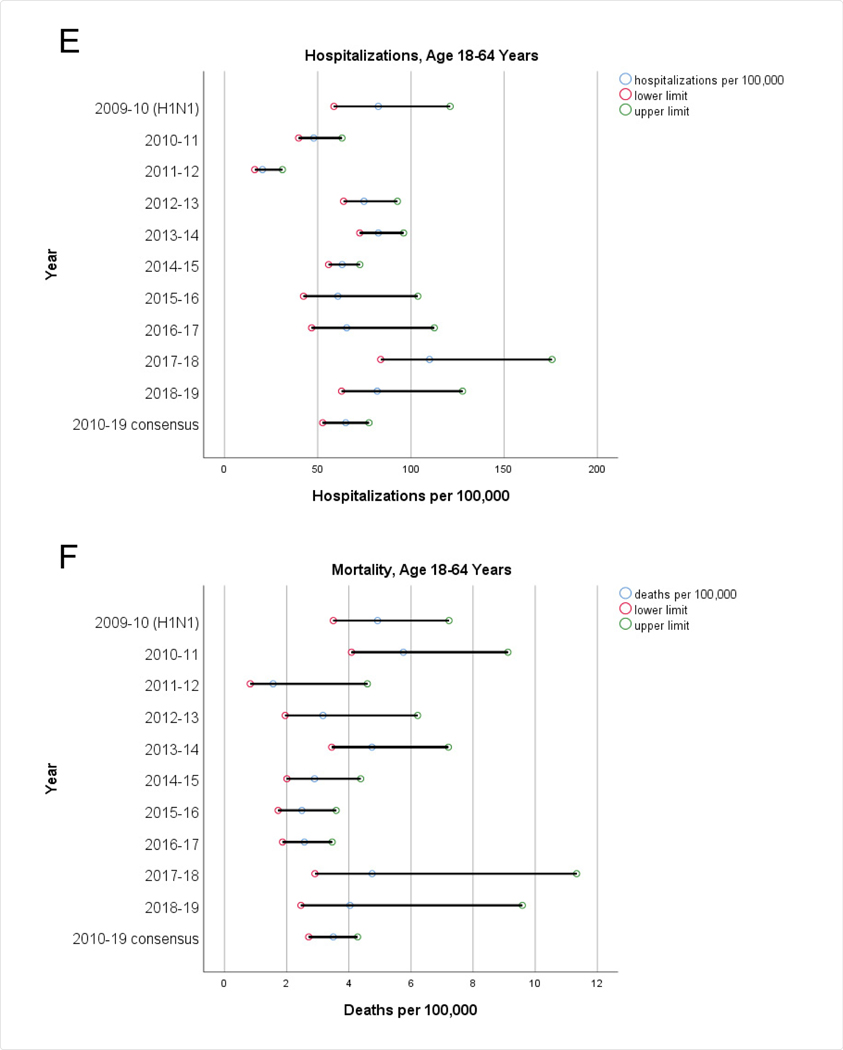

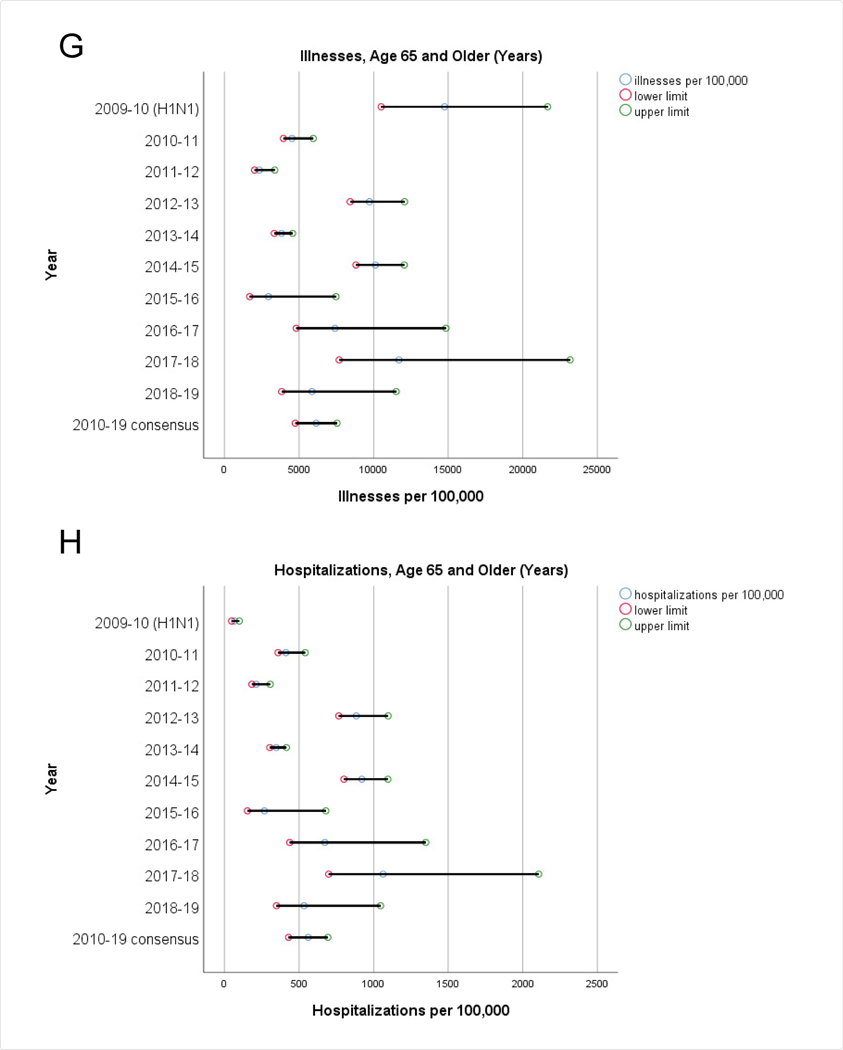

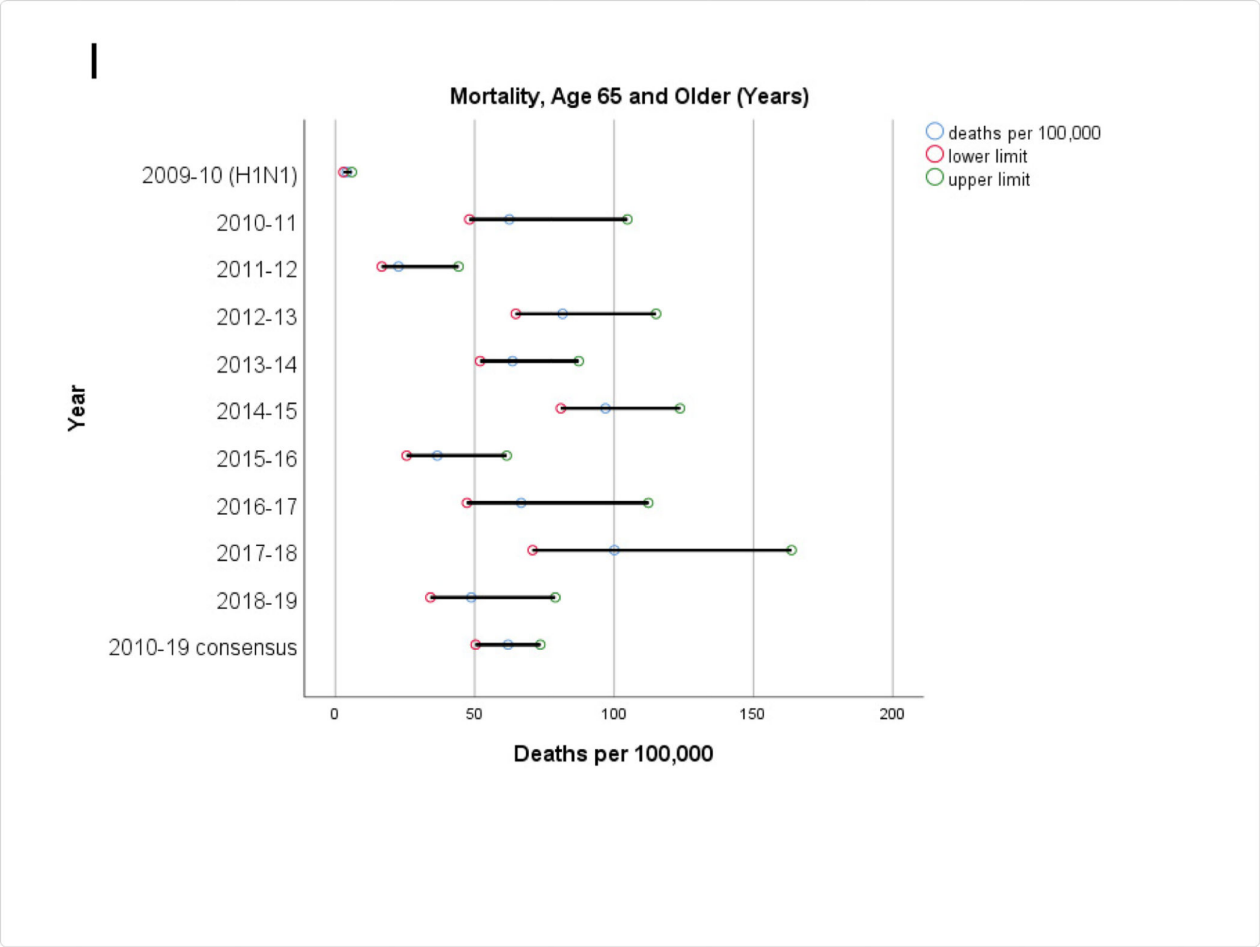
Rates of illnesses, hospitalizations, and deaths attributable to the influenza A (H1N1) pandemic (2009–10) [4] and seasonal influenza (2010–19) [1], by age group (A-C: 0–17 years; D-F: 18–64 years; G-I: 65+ years). The lower and upper limits represent estimated ranges for the 2009–10 through 2018–19 years. The lower and upper limits for the 2010–19 consensus values are 99% confidence intervals for the overall estimate, determined from a beta-binomial regression model [2,11,12] for the 2010–11 through 2018–19 years.

We show the age-specific rates of illnesses, hospitalizations, and deaths in Figure 2, along with summary measures of the rates over 2010–19, from a beta binomial random effects model. We also depict the corresponding rates from the 2009–2010 influenza A (H1N1) pandemic [4]. Estimated ranges are also shown for the yearly age-specific rates, and 99% confidence intervals are given for the summary consensus rates.

**Figure 3:**
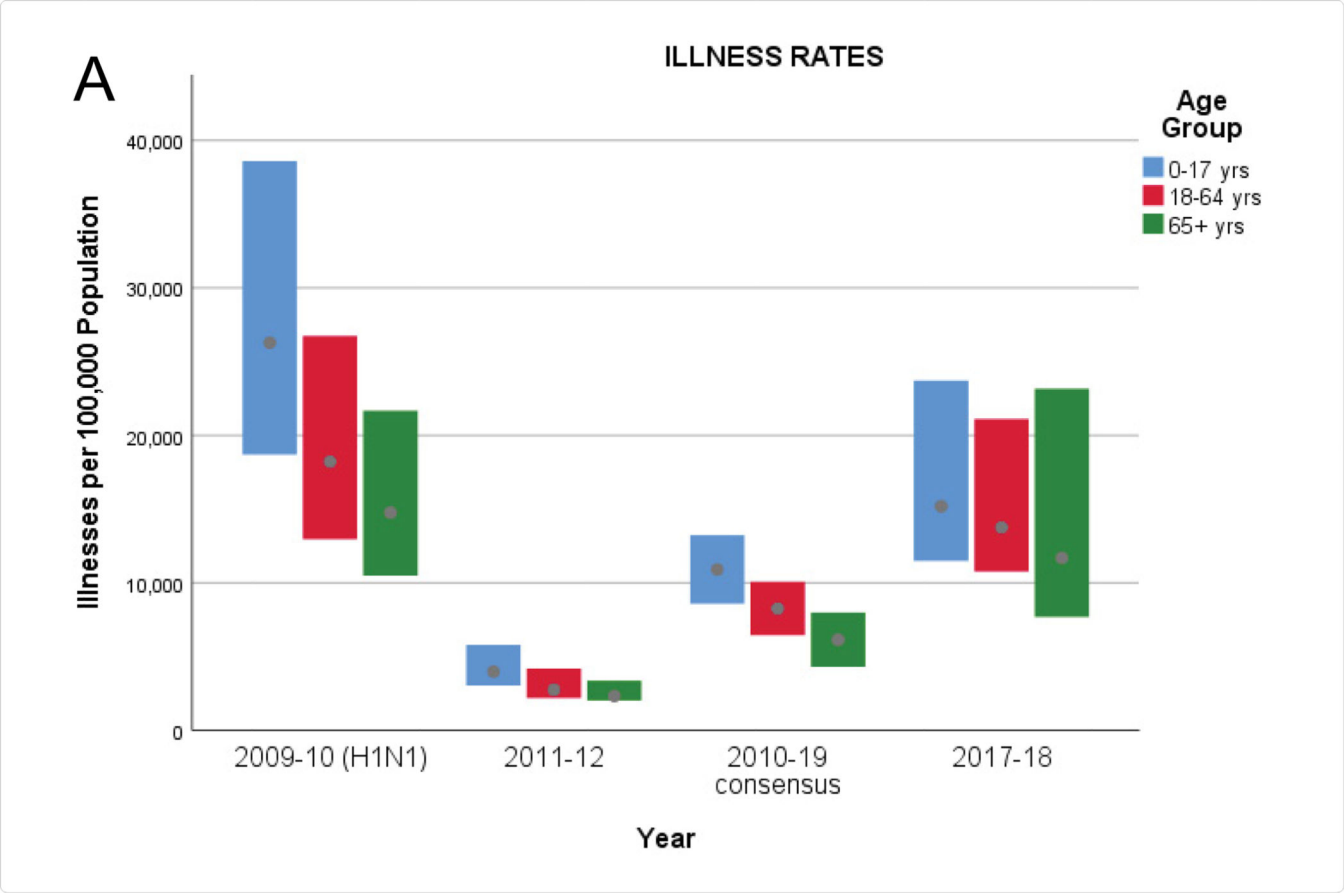

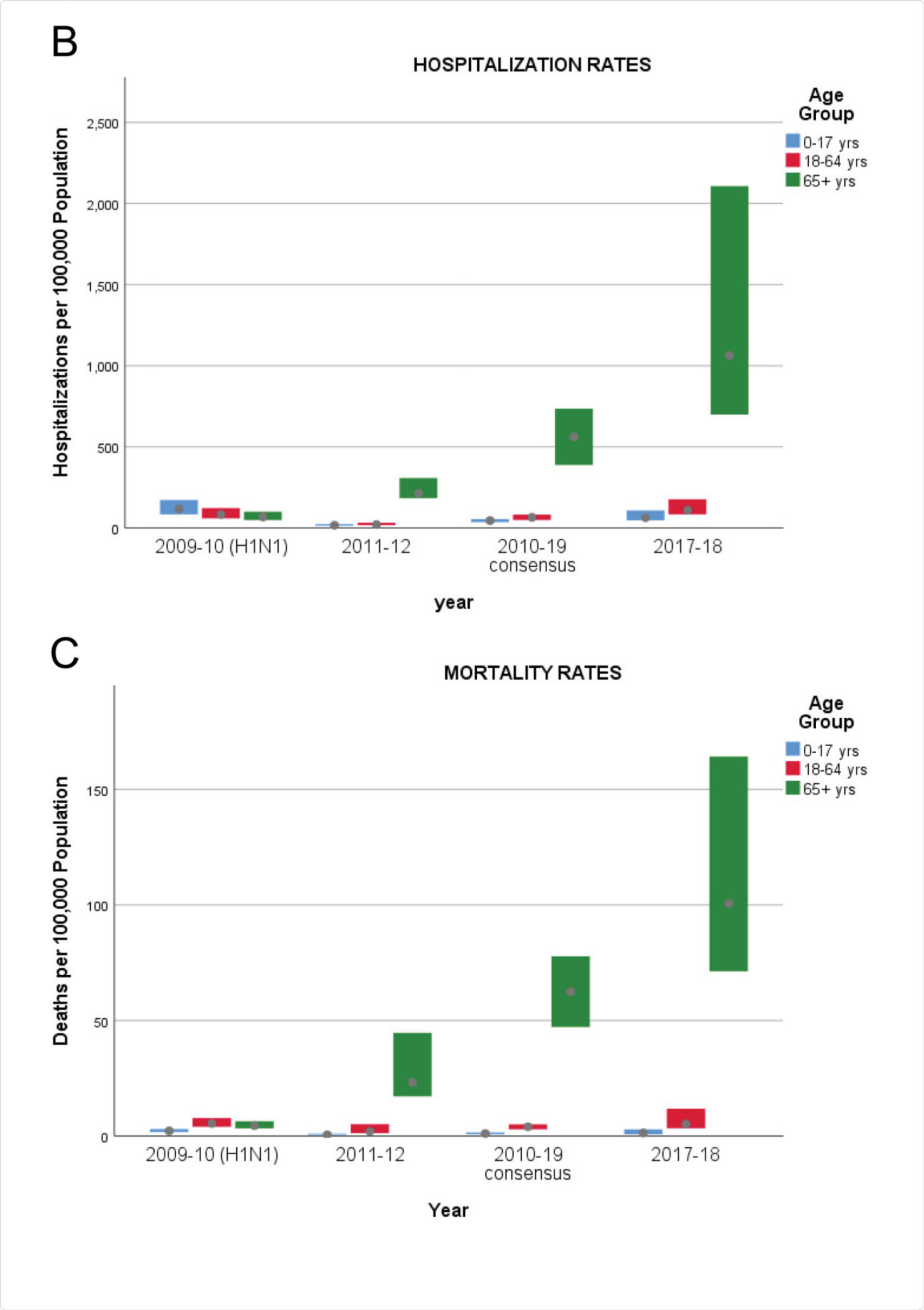
Range charts of rates of illnesses (A), hospitalizations (B), and deaths (C) attributable to the influenza A (H1N1) pandemic (2009–10), seasonal influenza for 2011–12 and 2017–18, and consensus values for 2010–19, by age group (0–17 years, 18–64 years, and 65+ years).

Lastly, we abstracted in Figure 3 the rates and ranges from Figure 2 corresponding to the 2009–10 influenza A pandemic, the consensus values for 2010–19, and the individual rates from 2011–12 ( a “low impact” flu season, from Figure 1) and 2017–18 (a “high impact” flu season, from Figure 1), to facilitate comparisons across different age groups.

## Discussion

Patterns in the annual rates of illnesses, medical visits, hospitalizations, and deaths attributable to seasonal influenza according to age are revealing (Figure 1). Clearly, the overall health impact varies by year, but relative differences between age groups are fairly stable. Rates of illnesses and medical visits are highest in infants age 0–4 years, followed by adults age 50–64 years. One might conjecture that infants naive to novel influenza viruses would be highly susceptible to illness, but recovery would typically be expected. Rates of hospitalizations and deaths evince a starkly different pattern, both dominated by elderly adults age 65 and over. Vulnerability in this age group is probably exacerbated by pre-existing health conditions, so avoidance of initial infection would be a prudent strategy.

How does the influenza A (H1N1) pandemic of 2009–2010 compare to the subsequent “normal” influenza seasons? Rates of illnesses during the H1N1 pandemic (2009–10) were about 2.4 times greater than the consensus rates for seasonal influenza (2010–19) across all age groups. For youths aged 0 to 17, the H1N1 hospitalization rate was about 2.6 times greater than the consensus rate for seasonal influenza, and the mortality rate was 2.9 times higher. One might thereby infer that influenza A (H1N1) is intrinsically more severe among youths than seasonal influenza. Among adults aged 18 to 64, hospitalization rates and mortality rates from H1N1 were 1.3 and 1.4 times greater respectively than the corresponding consensus rates for seasonal influenza. On the other hand, the hospitalization and mortality rates for elderly adults (aged 65 and older) during the H1N1 pandemic appear anomalous at a mere one-tenth the corresponding consensus rates for seasonal influenza, and their corresponding ranges seem disproportionately small. In light of subsequent findings, we conjecture that hospitalizations and deaths (and their spread) in this age cohort during the H1N1 pandemic were underestimated; in particular, one might expect that the hospitalization and mortality rates would be higher than in the younger adults aged 18 to 64. It is therefore doubtful that the influenza A pandemic of 2009–10 is a suitable model for subsequent influenza epidemics relative to the experience of elderly adults.

An important limitation of this retrospective analysis is the absence of information on other potential population characteristics and risk factors, such as gender and comorbidities, which likely affect disease morbidity and mortality. And, as noted above, possible under-detection of morbidity and mortality during the H1N1 pandemic in elderly adults might lead to erroneous inferences in highlighting specific at-risk populations or focusing preventive measures toward them.

## Conclusions and Implications

There are two pertinent take-home messages from our findings.

First, it is often of interest to establish a baseline assessment, that is, a typical influenza mortality profile, from the 2010–19 decade, as would be done prior to calculations of excess mortality. Our findings inject a note of caution into this endeavor. Selection of individual years to establish a baseline could be highly misleading: as an extreme example, the mortality profile of 2013–14 substantially under-represents the mortality profile of the decade, and the mortality profile of 2017–18 substantially over-represents the mortality profile of the decade. We found a significant linear trend in influenza mortality over the decade, and suggested that a regression approach adjusting for this trend would provide a reasonable consensus estimate of baseline mortality. But this is an a posteriori adjustment; prospectively, we have little reason to presume any adjustment would be needed.

Second, the disease burden of the 2009–10 influenza A pandemic was strikingly unlike that observed in the subsequent influenza seasons 2010 to 2019, in the United States: In particular, there was substantial negative excess influenza mortality among the elderly in 2009–10 compared to the subsequent decade. This is altogether surprising, especially since there was considerable mortality among the elderly during the 1918 pandemic, also attributed to the H1N1 strain [10]. The CDC methodology for assessing influenza burden in the United States is well-established [2]; nevertheless, the assumptions leading to the 2009–10 estimates relating to elderly mortality [5] ought to be scrutinized more closely.
